# Access and barriers to treatment and counseling for postpartum women with and without symptoms of (CB-)PTSD within the cross-sectional study INVITE

**DOI:** 10.1186/s12884-026-08660-x

**Published:** 2026-01-24

**Authors:** Lara Seefeld, Valentina Jehn, Julia Schellong, Susan Garthus-Niegel

**Affiliations:** 1 Department of Psychology & Neuroscience, Dalhousie University, Halifax, Canada; 2 Department of Psychotherapy and Psychosomatic Medicine, Faculty of Medicine, University Hospital Carl Gustav Carus, TUD Dresden University of Technology, Dresden, Germany; 3 Institute and Policlinic of Occupational and Social Medicine, Faculty of Medicine, TUD Dresden University of Technology, Dresden, Germany; 4 Institute for Systems Medicine (ISM), Faculty of Medicine, Medical School Hamburg MSH, Hamburg, Germany; 5 Department of Childhood and Families, Norwegian Institute of Public Health, Oslo, Norway

**Keywords:** Childbirth-related PTSD, Postpartum PTSD, Likelihood of help-seeking, Treatment barriers, INVITE study

## Abstract

**Background:**

Although childbirth is frequently described as a positive experience, the transition to parenthood can be challenging and many postpartum women suffer from mental health problems. Nevertheless, the utilization of treatment and counseling services remains low. To improve this, it is necessary to examine the likelihood of and barriers to help-seeking among postpartum women. There is a paucity of research on postpartum posttraumatic stress disorder (PTSD), that addresses these issues, especially when considering both types of postpartum PTSD, namely childbirth-related PTSD (CB-PTSD) and general PTSD (gPTSD). Thus, we examined differences in the likelihood of and barriers to help-seeking between postpartum women with CB-PTSD, gPTSD, and women who were not affected by clinically relevant symptoms of these two mental health problems.

**Methods:**

Data from the cross-sectional study INVITE were used, consisting of *n* = 3,875 telephone interviews with women between 6 weeks and 6 months after childbirth. CB-PTSD was assessed using the City Birth Trauma Scale and gPTSD using a short version of the Primary Care Posttraumatic Stress Disorder Screen for DSM-5. Women were asked about their likelihood of and specific barriers to help-seeking through self-developed questionnaires. Analyses of covariance were conducted to examine group differences and multiple regression analyses to investigate associations between symptom severity and the likelihood of help-seeking.

**Results:**

Groups did not differ in their likelihood of help-seeking. Additionally, the severity of the symptoms did not predict the likelihood of help-seeking. Post-hoc comparisons revealed more overall barriers among women with CB-PTSD compared to non-affected women. Concerning different types of barriers, women affected by CB-PTSD or gPTSD reported more instrumental barriers than non-affected women. More barriers related to fears about treatment and stigmatization were only reported by women with CB-PTSD compared to non-affected women but not by women with gPTSD. Significant associations with socio-demographic confounders were found in all analyses.

**Conclusions:**

Reducing instrumental barriers, e.g. through outreach services or financial subsidies, seems to be key in order to improve access to services for postpartum women with CB-PTSD or gPTSD. Since women with CB-PTSD reported more fears about treatment and stigmatization, education about CB-PTSD among perinatal women and health professionals may also be important.

**Supplementary Information:**

The online version contains supplementary material available at 10.1186/s12884-026-08660-x.

## Background

 Childbirth is often described as a positive, life-changing experience. However, the transition to parenthood is also full of challenges. Around 20% of women suffer from mental health problems during pregnancy or the postpartum period [1]. Among those, postpartum depression (PPD) and postpartum anxiety disorders (PAD) are the most prevalent and studied [2–5]. Other mental disorders are easily overlooked, as it is often the case with posttraumatic stress disorder (PTSD) in the postpartum period [6–8]. Due to the overlap of symptoms of negative mood and cognitions, postpartum PTSD is often misdiagnosed as PPD, potentially resulting in suboptimal treatment approaches [9].

### Specificity of PTSD in the postpartum period

Childbirth can function as a traumatic event that might evoke symptoms of PTSD in the postpartum period, which is mostly referred to as childbirth-related PTSD (CB-PTSD) [10, 11]. Between 9 and 44% of postpartum women perceive their childbirth as traumatic [12], among others due to objective obstetric factors such as birth complications and the mode of delivery [13, 14] but also due to subjective factors such as perceived massive stress and lack of control during childbirth [15–18]. Moreover, childbirth can function as a trigger reactivating symptoms of PTSD due to an event other than the recent childbirth [19]. Nevertheless, not all women with a traumatic childbirth appraisal develop clinically relevant CB-PTSD [20]. A recent meta-analysis estimated that 4.7% of postpartum women are affected by clinical CB-PTSD and 12.3% are affected by subclinical CB-PTSD [21]. Most postpartum women affected by symptoms of CB-PTSD recover spontaneously within the first six months [22], but there is a significant minority who suffers from CB-PTSD symptoms even two years later [23–25]. CB-PTSD is associated with negative consequences for the physical, social, and emotional wellbeing of mothers [26, 27], a decreased desire for having further children [28] difficulties in the couple relationship [29, 30], negative effects on breastfeeding [31], and adverse influences on mother-child bonding [32], early child development, and other child outcomes [24, 25, 33, 34].

**Fig. 1:**
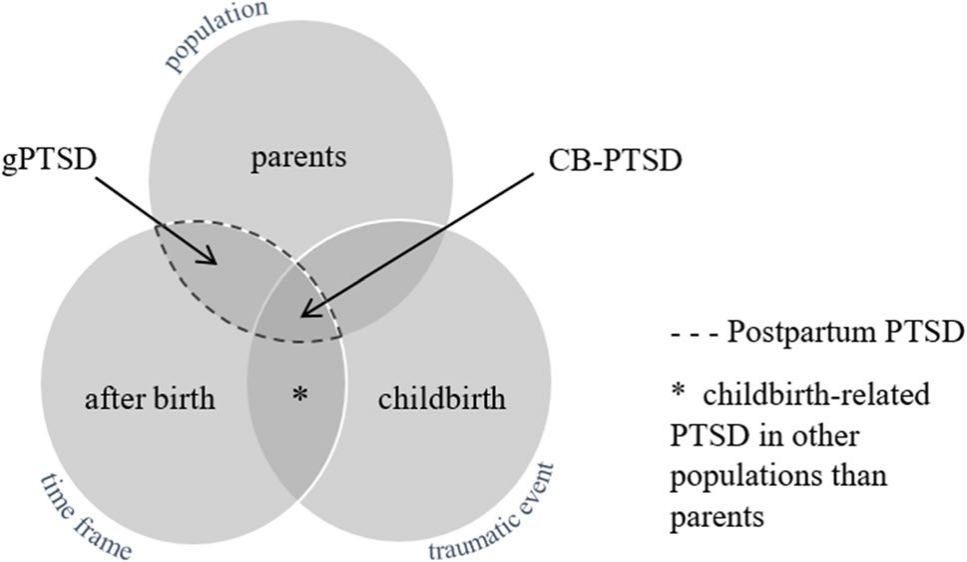
Terminology of postpartum PTSD. Adapted from Heyne et al. [21] by Harder et al. [36]. Reprinted with permission

In addition to CB-PTSD, there is a second layer of PTSD in the postpartum period, which is often neglected and difficult to distinguish from CB-PTSD: general PTSD among postpartum women (gPTSD), caused by another stressor unrelated to the recent childbirth [21]. Although CB-PTSD and gPTSD share all symptom clusters of PTSD, and the only difference is the A-criterion (traumatic childbirth for CB-PTSD vs. another traumatic event for gPTSD), there is some research suggesting that women with CB-PTSD show more re-experiencing compared to women with gPTSD, who show more avoidance symptoms [35]. Figure 1 illustrates the differentiation of the terms CB-PTSD and gPTSD.

Research about postpartum PTSD should include these different subtypes to ensure that this distinction is also considered in practical matters, e.g. when implementing disorder-specific treatments for CB-PTSD or gPTSD. To date, there are not many studies that consider these two subtypes of postpartum PTSD, in part because of difficulties in differentiating between the two specific disorders [37]. Often, the presence of PTSD symptoms in the postpartum period leads to the conclusion that they were caused by childbirth, ignoring other potentially traumatic events [38]. In a population-based maternity survey in England focusing on CB-PTSD or gPTSD in the postpartum period, 1/4 of the women affected by postpartum PTSD stated that childbirth had been the index event whereas 3/4 named a different event [35]. However, this is not reflected in the amount of research focusing on CB-PTSD rather than gPTSD in the postpartum period.

### Likelihood of help-seeking among postpartum women

Despite of the mentioned far-reaching negative consequences of postpartum PTSD and other mental health problems in the postpartum period, postpartum women are less likely to seek help for any type of psychological distress than women who have not given birth in the past year [39]. Thus, not all postpartum women experiencing mental health problems, including postpartum women with symptoms of PTSD, seek help in the form of treatment and counseling services [40], despite the existence of several effective treatment methods [41–46]. In order to understand this treatment gap, it is necessary to examine the help-seeking behavior of postpartum women affected by CB-PTSD and/or gPTSD^1^. This target group has specific characteristics that distinguish it from other PTSD-affected groups. For example, in the case of CB-PTSD, the traumatic index event childbirth is usually valued positively by society in contrast to other trauma events, such as sexualized or physical violence [11, 47].

Help-seeking as a problem-focused, intentional decision making process [48] has been studied several times among perinatal women, but mostly in the context of PPD [49–53] or as part of a broader approach without specific consideration of CB-PTSD or gPTSD [54, 55]. Studies on help-seeking of women with postpartum PTSD are lacking, and so is knowledge about their specific needs. However, this knowledge is indispensable to develop and implement specific, effective services in the treatment of postpartum women affected by CB-PTSD and/or gPTSD.

### Symptom severity and the influence on the likelihood of help-seeking

An important factor that may facilitate or hinder help-seeking is the symptom severity of the mental health problems in question. In a sample of individuals from the general population – as opposed to a sample of exclusively postpartum women – it was shown that the higher the PTSD-related distress, the more likely they were to seek professional help [56]. Studies examining postpartum women affected by PPD found the same association of higher symptom severity and higher help-seeking behavior [49, 52]. To the best of our knowledge, studies examining how symptom severity of CB-PTSD and/or gPTSD affects the likelihood of help-seeking behavior of postpartum women have not yet been conducted [57].

### Barriers to help-seeking

To improve access to counseling and treatment services for postpartum women affected by CB-PTSD and/or gPTSD, it is important to examine specific barriers that impede the process of seeking help. There is already a great deal of research on perceived barriers of postpartum women in the process of help-seeking, but mainly with a focus on women affected by PPD or PAD [50, 51, 58–60]. Specific barriers to perinatal mental healthcare mentioned in a conceptual framework include individual level barriers like the fear of being negatively judged by others or missing knowledge about mental health problems that leads to a non-recognition or misinterpretation of symptoms [61]. Additionally, women perceive instrumental barriers (e.g. no available services in the vicinity) that could hinder their help-seeking behavior [61].

Previous research shows that people affected by PTSD perceive more barriers than those affected by other mental health disorders [62]. Thus, women affected by postpartum PTSD may perceive more barriers than postpartum women with other mental health problems (e.g. PPD or PAD). Since postpartum women with CB-PTSD experience additional trauma-specific barriers (e.g. do not want to talk about the trauma since childbirth is valued positively in society [11]), they might report an even higher number of perceived barriers compared to women with other mental health problems or compared to women affected by gPTSD. The barriers of help-seeking of postpartum women with CB-PTSD and/or gPTSD may also differ in terms of content.

A parallel consideration and a comparison of postpartum women affected by CB-PTSD and postpartum women affected by gPTSD allows a group-specific identification regarding the number and type of barriers in the context of help-seeking behavior. We expected differences between the groups of women with CB-PTSD and/or women with gPTSD, as the traumatic index events are judged differently by society, which might particularly affect barriers concerning stigma. Moreover, the groups of women with CB-PTSD and/or gPTSD were expected to report more barriers than women who are not affected by clinically relevant symptoms of these two mental health problems. Comparing the help-seeking behavior of postpartum women with CB-PTSD and/or gPTSD can help to investigate whether, despite similar symptom expression, there are disorder-specific differences in the perception of barriers to help-seeking that need to be considered when developing and implementing treatment and counseling services.

### Objectives of the current study

The following research objectives were examined: (1) Potential differences in the likelihood of seeking treatment and counseling among women with CB-PTSD and/or gPTSD and women without clinically relevant symptoms of the mentioned mental health problems will be investigated exploratively. (2) It was be examined whether the severity of the symptoms predicts the likelihood of help-seeking. Due to no disorder-specific research regarding this association in the postpartum period, this was tested in an exploratory manner for both CB-PTSD and gPTSD. (3) It was investigated how postpartum women with CB-PTSD and/or gPTSD, and women without clinically relevant symptoms differ in the number and the type of possible barriers.

## Methods

### Design

In order to investigate the above-mentioned research objectives, data from the cross-sectional study INVITE were used. The aim of this study was to collect information about postpartum women affected by PPD, PAD, (CB-)PTSD, and intimate partner violence (IPV) and their preferences for and barriers to treatment and counseling in and around Dresden, Germany. Data were gathered via telephone interviews conducted by trained student assistants. All approached women who had sufficient German or English language skills, and who had given birth between 6 weeks and 6 months previously could participate, as this marks the end of the subacute postpartum period [63]. The recruitment process included all maternity hospitals and most birth centers in Dresden and is further described in the study protocol of the INVITE study [64]. After the approximately one-hour long telephone interview, all women were offered a list of appropriate treatment and counseling services in Dresden. As an incentive for participation all women received a small chocolate and 20€.

Data collection and management was facilitated using Research Electronic Data Capture (REDCap), a secure, web-based application for data capture as part of research studies, hosted at the “Koordinierungszentrum für Klinische Studien” at the Faculty of Medicine of the Technische Universität Dresden [65, 66].

### Sample

**Fig. 2:**
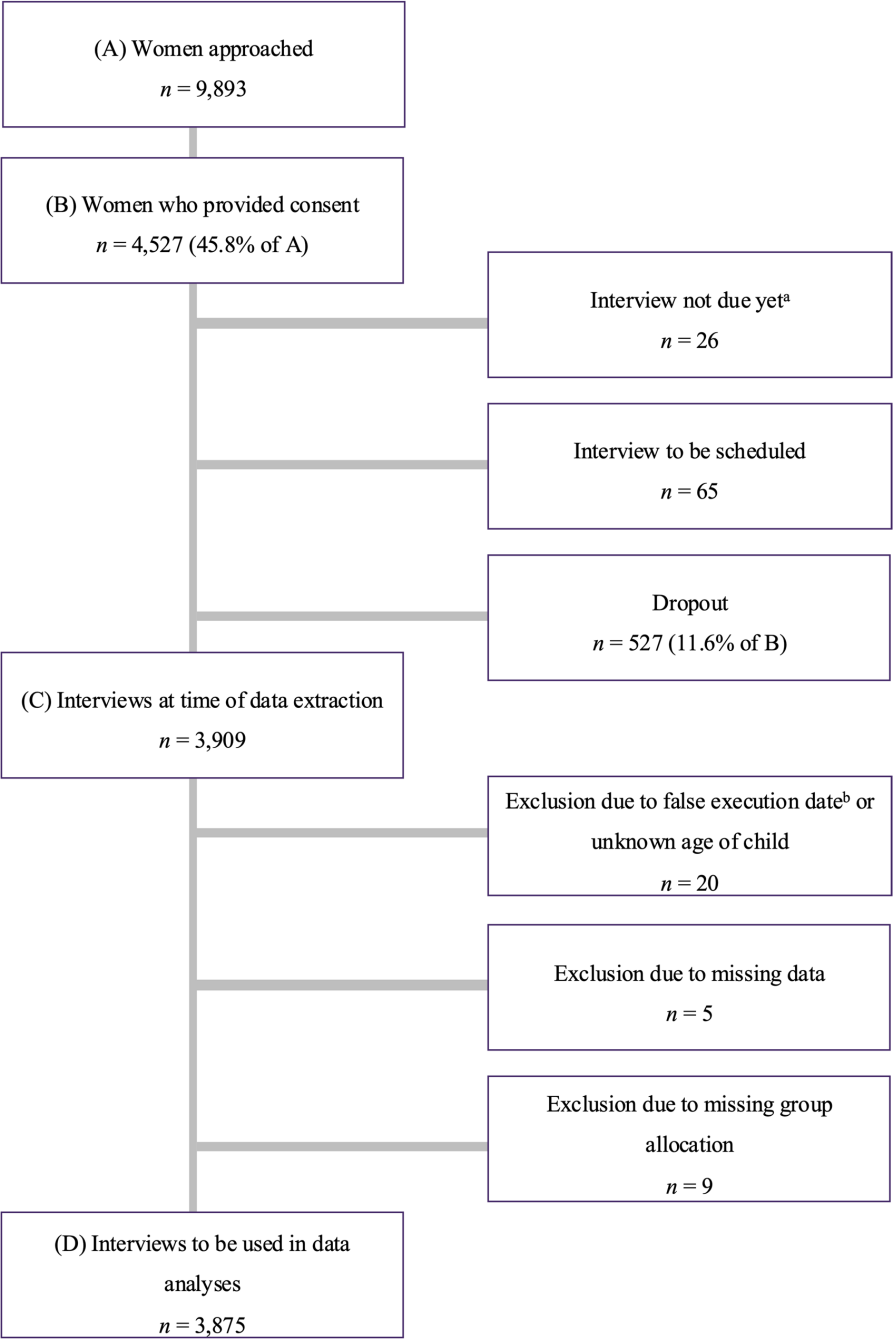
Flowchart of the current study. Note. The flowchart includes the response rate, dropouts, exclusions, and final sample size based on the recruitment process between November 2020 and July, 7th, 2023. **a** INVITE is ongoing. **b **Interviews were conducted outside the predefined period between 6 weeks and 6 months postpartum

For the current work, all data from the starting point of the study in November 2020 until July 7th 2023 were included. During this time frame, 9,893 women were approached, of whom 4,527 (45.8%) signed written informed consent to participate in the study, see Fig. 2. As the INVITE study was still ongoing when the current dataset was downloaded, some of these women (*n* = 91) had not yet been interviewed, because they were not yet in the time frame of at least 6 weeks after childbirth (*n* = 26) or because they had not yet been reached for the appointment (*n* = 65). In addition, 527 women dropped out of the study because they could not be reached for the interview within 6 months after childbirth or revoked their consent. Interviews conducted before 6 weeks or after 6 months postpartum or with missing information regarding the date of childbirth were excluded from further analyses (*n* = 20). Furthermore, data from 5 participants could not be included in the analyses due to missing data in all of the variables of interest and 9 women were excluded due to missing values in group specific items, and thus missing group allocation. The final sample used in the statistical analyses comprised *N* = 3,875 women.

### Measures

Standardized and validated quantitative instruments as well as newly constructed questionnaires supplemented with additional questions (e.g., demographic and socioeconomic factors, past and current mental health problems and treatments) were used to examine the constructs of interest.

#### City birth trauma scale (City BiTS)

To screen for CB-PTSD, the German version of the City BiTS, a 22-item questionnaire developed to measure CB-PTSD according to DSM-5 criteria, was used [67, 68]. Participants answered two dichotomous items (“yes/no”) about the possible existence of the stressor criterion childbirth (i.e., threat of death or serious injury to the mother or baby during labor, delivery, or immediately thereafter). Additional items about specific symptoms of CB-PTSD (intrusions, avoidance, negative cognitions/mood, and hyperarousal) and their frequency over the last week were scored on a 4-point Likert scale ranging from 0 (“not at all”) to 3 (“5 or more times”) to yield a total score of 0 to 60, with higher scores indicating a higher level of CB-PTSD symptoms and thus a higher severity of the symptoms of CB-PTSD [67]. In order to take the CB-PTSD exclusion criteria into account, women were asked about symptom onset, distress, everyday impairments and other causes of symptoms. Cronbach’s alpha indicated good reliability (α = 0.84) in our sample.

#### Primary care posttraumatic stress disorder screen for DSM-5 (PC-PTSD-5)

To assess symptoms of gPTSD among postpartum women that were not caused by childbirth as the index event, a modified version of the PC-PTSD-5, an instrument to screen for PTSD according to DSM-5 criteria [69] was used. In the context of the INVITE study, the instructions of the PC-PTSD-5 were modified so that all participants were asked to think about an event that was “so frightening, horrible, or upsetting” that they may have experienced any of the five listed PTSD related symptoms (nightmares about the event, avoidance behavior with regard to trauma-relevant stimuli, generally increased arousal, feelings of alienation, and feelings of guilt about the event during the past month) [35, 64]. Women could answer each of these five items with “yes”/”no” resulting in a PC-PTSD-5-score ranging from 0 to 5 with a Cronbach’s alpha of 0.73. Another modification to the original instrument was that if women answered “yes” to any of the five items, they were subsequently asked how stressful they experienced this symptom on a 5-point-Likert scale from 0 (“not at all”) to 4 (“extremely stressful”). To assess symptom severity, the five items of the perceived stress experience were summarized to a gPTSD symptom severity scale ranging from 0 to 20, with higher scores indicating higher stress experience and thus higher symptom severity of gPTSD. To ensure that the reported symptoms related to a traumatic event outside of childbirth, a trauma list with seven exemplary events and a free text option was presented (e.g. serious life-threatening illness, physical assault). In a multiple response format, women were asked to indicate which of these event(s) they had experienced and only symptoms of women with a traumatic event other than childbirth were counted as gPTSD symptoms.

#### Likelihood of seeking treatment and counseling

Various counseling and treatment services for postpartum mental health problems were mentioned to the women and, if unknown, described in order to determine their preferences. These services included (1) Professional and Personal Confidants (e.g., midwife or woman in the same situation), (2) Communal and Psychosocial Services (e.g., household help or psychosocial crisis service), (3) Medical Services (e.g., general practitioner or pediatrician), (4) Psychotherapeutic Services (e.g., day clinic for psychiatry or psychosomatic medicine or specialized trauma outpatient clinic). All women, with or without mental health problems, were asked to indicate on a 11-point Likert scale from 0 (“very unlikely”) to 10 (“very likely”) how likely they would seek treatment or counseling if they would be (or are) affected by symptoms of PPD, PAD, and/or (CB-)PTSD.

#### Barriers to Help-Seeking

To assess potential barriers to treatment and counseling a self-generated questionnaire was developed [64] based on literature regarding the Health Beliefs Model [70]. All women were asked to rate how likely it is that the barriers would keep them from seeking help using a 5-point Likert scale, ranging from 0 (“not true at all”) to 4 (“very true”). The sum score of 15 items was used for the number of barriers and revealed a good reliability in our sample (α = 0.80). Additionally, items were categorized according to Seefeld et al. [71, unpublished observation] into the following three subscales of barriers to help-seeking among postpartum women: (1) Fears about Treatment and Stigmatization (e.g. “I would be scared because I don’t know what takes place during counseling or treatment”), (2) Health Beliefs (e.g. “I don’t think talking to a specialist about my problems would help me”), and (3) Instrumental Barriers (e.g. “I wouldn’t have time for counselling or treatment”). Each subscale comprised 5 items and subscale scores ranged from 0 to 20. Cronbach’s alpha in our sample was 0.84 in Subscale (1), 0.64 in Subscale (2), and 0.58 in Subscale (3).

### Data analysis

IBM SPSS Statistics (version 29.0.0.0) was used for all statistical analyses. Four different groups were computed: women with CB-PTSD, women with gPTSD, women with comorbid symptoms, and women without clinically relevant symptoms of CB-PTSD or gPTSD (non-affected). The first group (CB-PTSD) included women who met the relevant DSM-5-criteria of CB-PTSD assessed by the City BiTS. As the need for the birth related index event (A criterion) of CB-PTSD has been critically discussed during the development of the definition of PTSD symptoms in DSM-5 [71, 72], and because it is already known that even an objectively uncomplicated birth can be subjectively perceived as heavily stress causing without experienced threat of death or serious injury to the mother or baby [15, 73], fulfillment of the A criterion was not required for group assignment to CB-PTSD in this study. The second group (gPTSD) contained women with a PC-PTSD-5-score ≥ 4 [74] and with a specified traumatic event not related to childbirth. The comorbid group included women who met conditions of both the CB-PTSD and the gPTSD group. The last group (non-affected) contained all women without any clinically relevant symptoms of the two screened disorders.

Based on previous literature revealing the impact of specific sociodemographic variables on the likelihood of and specific barriers to help-seeking among postpartum women with general mental health problems [51, 75–77] and associations of specific sociodemographic characteristics with the amount of barriers in a previous study [[71], unpublished observation], measures of association (Pearson correlation coefficients and eta) between the predictors and outcomes of the respective analyses and the following variables were calculated: maternal age, duration of residence in Germany, income, and number of children. Variables with a significant association (*p* <.05) with the outcome variable (likelihood of help-seeking and sum score of barriers) were included in the respective analyses as covariates.

The relevant data were checked for assumptions for all analyses. If there were missing values of less than 20% in one scale they were replaced by the woman’s mean value. Descriptive analyses were computed for all relevant variables in terms of mean and standard deviation and data were checked for outliers. An analysis of covariance (ANCOVA) was used to compare the mean score of the likelihood of help-seeking between the mentioned groups including identified confounding variables as covariates. Two multiple linear regression analyses (MLRs) were used to determine whether the severity of symptoms of CB-PTSD or gPTSD predicted the likelihood of help-seeking including relevant confounders. Values whose studentized excluded residuals were less than − 3 or greater than 3 were considered outliers and excluded. In the MLR for the symptom severity of CB-PTSD, this applied to 55 cases, in the MLR for the symptom severity of PTSD to 11 cases. Both MLRs were performed with bootstrapping using the bias-corrected and accelerated (BCa) method with 95% confidence intervals (CI) and 1,000 iterations. A second ANCOVA was then computed to examine the differences in the barrier sum score between all groups to reveal differences in the number of perceived barriers.

To examine the differences between the groups in their subscale scores of the barriers, a multivariate analysis of covariance (MANCOVA) was computed. There were 25 outliers (2 univariate outliers and 23 multivariate outliers) that were excluded from the dataset. The number of included women varied slightly between the analyses due to missing values in individual variables. In the case of significant group differences (*p* <.05), post hoc tests were conducted using Bonferroni-corrected mean comparisons based on the estimated marginal means.

Covariates were not homogeneously distributed across the symptom groups. These violations of the homogeneity of the covariates are addressed in the discussion section.

### Power analysis

An a priori power analysis was computed using G*Power 3.1.9.7 to determine the required sample size. The power analysis for F-Tests (ANCOVA) indicated that a total sample of *n* = 244 women is needed to detect small effects (*d* = 0.2) with 80% (1-beta) power and alpha at 0.05. Other research questions were investigated through multiple linear regression analyses, for which the proposed sample size is below the above-mentioned sample size (results not shown). A power analysis for MANCOVA is not readily available via G*Power but based on the calculated sample size of a MANOVA (*n* = 88, *f*^*2*^ = 0.0625, 80% power, α = 0.05) and an expected sample size that is much larger than that, we assume good statistical power for the planned MANCOVA analyses.

## Results

### Descriptive statistics

The final sample included *N* = 3,875 women, whose descriptive characteristics are shown in Table 1. At the time of the interview, the women were on average 32.95 (*SD* = 4.64) years old and their youngest child was 13.17 (*SD* = 2.71) weeks old. Most women were born in Germany (91.2%), currently in a relationship (97.7%), and indicated a high level of education, which was defined as more than 10 years of education (74.0%). Due to the small sample size of the group of comorbid women (*n* = 15), this group was excluded from further analyses of group comparisons as the statistical power would be too low. However, the group of comorbid women was included for descriptive evaluations.

**Table 1:** Descriptive statistics of the sample

Sample characteristics	Total sample (*n* = 3875)
**Maternal age** ^a^	32.95 ± 4.64 (16.78–53.96)
**Child´s age** ^b^	13.18 ± 2.71 (6.00–26.00)
**Duration of residence in Germany** ^c^	
< 5 years	103 (2.7%)
5–10 years	115 (3.0%)
> 10 years	124 (3.2%)
Born in Germany	3533 (91.2%)
**Income** ^d^	
< 1.250	93 (2.4%)
1.250 - 2.249	398 (10.3%)
2.250 - 2.999	493 (12.8%)
3000 - 3999	1001 (25.9%)
4000 - 4999	960 (24.9%)
> 5000	914 (23.7%)
**Partnership status**	
Partner	3785 (97.7%)
No Partner	90 (2.3%)
**Education**	
≤ 10 years	1008 (26.0%)
> 10 years	2865 (74.0%)
**Parity**	
Primiparous	2030 (52.4%)
Multiparous ^e^	1845 (47.6%)
1	1415 (76.7%)
2	332 (18.0%)
3	74 (4.0%)
≥ 4	23 (1.2%)
**Current mental health problem** ^f^	
Yes	274 (7.1%)
No	3461 (89,3%)
Don’t know	140 (3,6%)
**Perception of birth as traumatic** ^g^	3.1 ± 2.9 (0–10)
non-affected	2.9 ± 2.8 (0–10)
CB-PTSD	6.9 ± 2.2 (0–10)
gPTSD	3.3 ± 3.0 (0–9)
Comorbid CB-PTSD & gPTSD	7.8 ± 1.8 (3–10)
**Likelihood of help-seeking**	7.8 ± 2.2 (0–10)
non-affected	7.8 ± 2.1 (0–10)
CB-PTSD	7.7 ± 2.2 (0–10)
gPTSD	7.9 ± 2.3 (0–10)
Comorbid CB-PTSD & gPTSD	7.4 ± 2.6 (2–10)
**Barriers to help-seeking**	31.5 ± 7.0 (15–60)
non-affected	31.3 ± 6.9 (15–60)
CB-PTSD	34.3 ± 7.1 (17–53)
gPTSD	33.7 ± 7.6 (17–51)
Comorbid CB-PTSD & gPTSD	34.1 ± 7.5 (20–47)

*Note. n* (%) or *M ± SD* (Range). City BiTS score = sum score of the City Birth trauma scale (0–60),)

^a^in years, ^b^in weeks, ^c^time since possible migration to Germany, ^d^in Euros per month and household, ^e^ additional children not relating to the last birth, ^f^based on self-report, ^g^ self-rated on a 11-point-Likert scale from 0 (“not traumatic at all”) to 10 (“very traumatic”, n varied slightly due to missing values in individual items

Women affected by CB-PTSD reported the highest barrier sum score (*M* = 34.3, *SD* = 7.1) compared to the groups of women with gPTSD, comorbid CB-PTSD and gPTSD, and non-affected women. Examining the different types of barriers to help-seeking among all women, the three highest rated and most common ones were: “I would rather talk to friends or family about my problems (*M* = 3.06, *SD* 1.032), “I would not have childcare.” (*M* = 2.66, *SD* = 1.122), and “I would not have time for counseling or treatment” (*M* = 2.51, *SD* = 1.028). Only 7.1% of all women reported current mental health problems (respectively 23.8% of women with CB-PTSD, 27.6% of women with gPTSD) and half of these women (53.8%) were currently receiving professional help (44.1% of women with CB-PTSD and 55.6% of women with gPTSD).

### Correlation analyses

Correlation analyses were conducted with all potential confounders and outcome variables to decide whether they should be included as confounding variables in the respective analyses. For the variables maternal age, duration of residence in Germany, and income statistically significant correlations were found with the outcome likelihood as well as with the outcome barrier sum score (*p* <.05). The same confounding variables showed significant correlations with the outcomes of the three barrier subscales, which were therefore included in the context of the MANCOVA. As the confounding variable number of children only correlated significantly with Subscale (3) Instrumental Barriers, it was not included in the MANCOVA (see Table 2).

**Table 2:** Association matrix of potential confounders and outcome variables

	1.	2.	3.	4.	5.	6.	7.	8.	9.
1. Maternal age	−								
2. Residence ^a^	0.072^***^	−							
3. Income	0.210^***^	0.062^***^	−						
4. No. children ^b^	0.324^***^	0.071^***^	0.035^*^	−					
5. Likelihood ^c^	**0.064** ^***^	**0.047** ^*^	**0.089** ^***^	−0.020	−				
6. Barrier sum	**−0.112** ^***^	**0.102** ^***^	**−0.105** ^***^	0.020	−0.341^***^	−			
7. Subscale 1^d^	−0.107^***^	0.057^***^	−0.061^***^	−0.021	−0.231^***^	0.833^***^	−		
8. Subscale 2^e^	−0.117^***^	0.086^***^	−0.090^***^	0.010	−0.415^***^	0.775^***^	0.500^***^	−	
9. Subscale 3^f^	−0.034^*^	0.103^***^	−0.098^***^	0.066^***^	−0.159^***^	0.722^***^	0.386^***^	0.334^***^	−

Note. Pearson correlation coefficients are shown for associations between two continuous variables and eta is shown for associations between ordinal (residence) and continuous variables. Significant associations between confounders and outcome variables (likelihood and barrier sum score) are printed in bold. Potential confounders = maternal age, duration of residence in Germany, income, and number of children. *n* varied slightly due to missing values in individual items

^a^duration of residence in Germany ^b^ number of children, ^c^likelihood of help-seeking, ^d^ Subscale 1: Fears about Treatment and Stigmatization, ^e^Subscale 2: Health Beliefs, ^f^ Subscale 3: Instrumental Barriers

*p < .05. **p < .01. *** *p* < .001

### Likelihood of help-seeking across the symptom groups

To identify differences between the groups of women affected by CB-PTSD, women affected by gPTSD, and women who are not affected by clinically relevant symptoms of these mental health problems with regard to the likelihood of help-seeking, an ANCOVA was carried out.^2^ After adjusting for the confounders, no statistically significant group difference was found in the likelihood of help-seeking, *F*(2, 3823) = 0.487, *p* =.615, partial η² = 0.000, see Table A1 in Appendix A. The likelihood of help-seeking differed with regard to the confounder maternal age *F*(1, 3823) = 7.729, *p* =.005, partial η² = 0.002, and income *F*(1, 3823) = 23.231, *p* <.001, partial η² = 0.006. However, the partial η² values indicated a very small effect size according to Cohen [78].

### Likelihood of help-seeking and the symptom severity of CB-PTSD and gPTSD

The relationship between symptom severity of CB-PTSD or gPTSD and the likelihood of help-seeking was tested using two multiple linear regression analyses with bootstrapping, including the confounders maternal age, duration of residence in Germany, and income. Neither the severity of CB-PTSD symptoms nor the severity of gPTSD symptoms predicted women’s likelihood of help-seeking (see Table A2 in Appendix A).

### Number of barriers to help-seeking across the symptom groups

The difference between the groups of women affected by CB-PTSD, women affected by gPTSD, and women who are not affected by clinically relevant symptoms of these mental health problems with regard to the number of barriers to help-seeking was examined using an ANCOVA. Maternal age, duration of residence in Germany, and income were added to the model as confounding variables. Moreover, the interaction term of group*maternal age was added due to the unmet statistical requirement of homogeneity of the regression slopes regarding the confounding variable maternal age. After controlling for these confounders and the interaction effect, the groups did not differ significantly in the barrier sum score, *F*(2, 3815) = 2.720, *p* =.066 partial η² = 0.001, see Table A3 in Appendix A.

### Subscales of barriers to help-seeking across the symptom groups

To examine differences between the groups of women affected by CB-PTSD, women affected by gPTSD, and women who are not affected by clinically relevant symptoms of these mental health problems on the subscales of barriers to help-seeking, controlling for the confounders maternal age, duration of residence in Germany, and income, a one-way MANCOVA was performed. A statistically significant difference between the symptom groups on the combined dependent variables was found, *F*(6, 7570) = 8.526, *p* <.001, partial η² = 0.007, Wilk’s Λ = 0.987. However, the symptom groups did not differ significantly in all subscales (see Table A4 in Appendix A): There were significant group differences in Subscale (1) Fears about Treatment and Stigmatization (*F*(2, 3787) = 12.468, *p* <.001, partial η² = 0.007) and in Subscale (3) Instrumental Barriers (*F*(2, 3787) = 17.978, *p* <.001, partial η² = 0.009). The effect sizes were both small according to Cohen [79]. There were no significant group differences in Subscale (2) Health Beliefs (*F* (2, 3787) = 1.105, *p* =.331, partial η² = 0.001).

To detect differences between the three groups (CB-PTSD, gPTSD, non-affected) in the two subscales, Bonferroni-corrected post hoc analyses were computed, see Table 3. In Subscale (1) Fears about Treatment and Stigmatization, women with CB-PTSD reported significantly more barriers than women without clinically relevant symptoms of CB-PTSD or gPTSD (*p* <.001). Also, women with CB-PTSD and women with gPTSD reported significantly more barriers in Subscale (3) Instrumental barriers than women without clinically relevant symptoms of CB-PTSD or gPTSD (*p* <.001).

**Table 3:** Differences of symptom groups in the subscales of barriers to help-seeking

(I) Comparing symptom groups	(J) Comparing symptom groups	Mean difference (I-J)	*p*	95% CI	
				LL	UL
Fears about Treatment and Stigmatization					
non-affected ^a^	CB-PTSD	**−1.241**	< 0.001***	−1.899	− 0.582
	gPTSD	− 0.775	0.063	−1.580	0.030
CB-PTSD	non-affected	**1.241**	< 0.001***	0.582	1.899
	gPTSD	0.466	0.824	− 0.555	1.487
gPTSD	non-affected	0.775	0.063	− 0.030	1.580
	CB-PTSD	− 0.466	0.824	−1.487	0.555
Instrumental Barriers					
non-affected	CB-PTSD	**−1.149**	< 0.001***	−1.708	− 0.589
	gPTSD	**−1.033**	< 0.001***	−1.717	− 0.349
CB-PTSD	non-affected	**1.149**	< 0.001***	0.589	1.708
	gPTSD	0.116	1	− 0.752	0.984
gPTSD	non-affected	**1.033**	< 0.001***	0.349	1.717
	CB-PTSD	− 0.116	1	− 0.984	0.752

Note. *CI* Confidence interval, *LL* Lower limit, *UL* Upper limit

^a^Postpartum women without clinically relevant symptoms of CB-PTSD or gPTSD. Significant group mean differences are printed in bold

*p < .05. **p < .01. *** *p* < .001

## Discussion

The aim of the study was to broaden the knowledge about help-seeking behavior of postpartum women affected by CB-PTSD and/or gPTSD in order to improve access to appropriate services. For this purpose, differences in the likelihood of and barriers to help-seeking between postpartum women with CB-PTSD, gPTSD, and women who were not affected by clinically relevant symptoms of these two mental health problems were examined.

There were no group differences in the likelihood of help-seeking among the groups of postpartum women with CB-PTSD, postpartum women with gPTSD, or postpartum women without clinically relevant symptoms of these two mental health problems. In addition, symptom severity of CB-PTSD or gPTSD did not predict the likelihood of help-seeking. An overall group-comparison of the number of possible barriers to help-seeking did not reveal any group differences. However, women affected by CB-PTSD and women affected by gPTSD reported more instrumental barriers compared to the non-affected women, and women affected by CB-PTSD reported more barriers regarding fears about treatment and stigmatization than non-affected women.

In our sample, 3.7% of the participants screened positive for CB-PTSD, 2.5% for gPTSD, and 0.4% for comorbid CB-PTSD and gPTSD. The prevalence of CB-PTSD is above the prevalence of 2.6% found in a comparable sample of postpartum women in Germany by Weigl et al. [67]. Contrary to that study, we decided against the necessary presence of the A criterion (i.e., threat of death or serious injury to the mother or baby during labor, birth or immediately afterwards) for group allocation to CB-PTSD. As a result, women who did not report an A criterion but still fulfilled all other relevant symptoms of clinical CB-PTSD (*n* = 61) were assigned to the CB-PTSD group. In this way, we were able to include more postpartum women, who were distressed due to CB-PTSD symptoms, and for whom access to appropriate services should be improved. Consequently, our CB-PTSD group has less strict inclusion criteria than in Weigl et al. [67] which may have led to the higher prevalence.

### Likelihood of help-seeking across the symptom groups

Contrary to our hypothesis, the groups of women with CB-PTSD, women with gPTSD, and non-affected women in our sample did not differ in terms of their reported likelihood of help-seeking. It is remarkable that the mean values of the scale assessing the likelihood of help-seeking were relatively high across all groups of postpartum women, because it means that regardless of whether or not women had symptoms of CB-PTSD or gPTSD, they rated their likelihood of help-seeking as relatively high. A high self-reported likelihood of help-seeking has already been found among the general population in Germany [80], when participants were asked how likely they would seek treatment or counseling if they would be (or were) affected by mental health problems.

A possible explanation for the high scores of the likelihood of help-seeking indicated by all postpartum women of our sample and a lack of group differences could be that positive attitudes towards mental health problems and psychotherapeutic treatment have increased substantially among the general population in Germany over the last years [81], thus including postpartum women. Positive attitudes of help-seeking are an important indicator of actual help-seeking for mental health problems, as shown in a study by Mojtabai et al. [82] based on a sample of the general population.

However, many women of our sample who were affected by CB-PTSD or gPTSD failed to translate their theoretically high likelihood of help-seeking into actual help-seeking as seen by the low number of women who were actually receiving help. Have et al. [80] suggest that this could be due to low values of the perceived effectiveness of treatment options. In addition, there are other barriers that impede the actual help-seeking process as discussed in a later section. Another explanation could be a lack of self-awareness of their symptoms. Many women who were affected by CB-PTSD or gPTSD did not report a mental health problem at all, although they were assigned to the group of women with CB-PTSD or gPTSD by validated screening instruments. Thus, it can be assumed that many affected women did not recognize their own symptoms as part of a mental health problem leading to no help-seeking behavior. This is consistent with findings of Daehn et al. [40], which indicate that a lack of self-awareness and identification of mental health problems among perinatal women leads to less help-seeking behavior. Similarly, Webb et al. [83] named lacking knowledge about mental health problems as one of the most widespread barriers to help-seeking behavior of postpartum women.

### Symptom severity of CB-PTSD or gPTSD as predictor of the likelihood of help-seeking

The severity of symptoms of CB-PTSD or gPTSD among postpartum women did not predict the likelihood of help-seeking in our sample.

In an unpublished study by Seefeld et al. [84] that focused on postpartum women and their likelihood of help-seeking depending on the severity of symptoms of PPD and/or PAD, a higher symptom severity of PPD predicted a lower likelihood of help-seeking. Conversely, a higher symptom severity of PAD predicted a higher likelihood of help-seeking. Our results indicate that a similar relationship between the symptom severity of CB-PTSD or gPTSD and the likelihood of help-seeking does not exist. Thus, in order to improve access to treatment and counseling for women affected by symptoms of CB-PTSD or gPTSD, focusing on other aspects besides disorder-specific symptom severity such as the reduction of barriers seems to be crucial as discussed in the later section about barriers to help-seeking.

### Barriers to help-seeking

No overall difference in the sum score of barriers across all symptom groups was found, but women differed slightly in their ratings of different types of barriers. This might indicate that women with CB-PTSD, gPTSD, and women without clinically relevant symptoms of postpartum PTSD do not differ in their total number of barriers to help-seeking, but instead in how much different types of barriers keep them from seeking help. Women affected by CB-PTSD reported more barriers in Subscale (1) Fears about Treatment and Stigmatization than women without clinically relevant symptoms of CB-PTSD or gPTSD. The pattern is different for women with gPTSD, who did not report more barriers in this subscale compared to non-affected women. Thus, women affected by CB-PTSD appear to have specific fears regarding treatment and concerns about stigmatization. Other studies have already shown that stigma and shame are widespread among postpartum women with general mental health problems [54, 61, 85, 86]. As described by Sonnenburg et al. [87] there is an ideal image of motherhood including expectations about fulfilling and self-sacrificing care for the child, which could cause psychological distress when women perceive discrepancies between this image and their own behavior. Thus, many postpartum women affected by mental health problems are afraid of being perceived as a bad mother and experience shame and fears about stigmatization [83]. In case of CB-PTSD, where childbirth is the traumatic index event and therefore the trauma and PTSD symptoms are directly related to the child, women may be even more reluctant to seek help than women with gPTSD, where the traumatic index event is not related to childbirth. This could explain why only women with CB-PTSD reported more barriers in this subscale compared to the group of non-affected women, whereas women with gPTSD did not. Another aspect of the difference regarding this subscale can be explained by results of Kingston et al. [88] that indicate that there is little knowledge about mental health problems of postpartum women among the general population. Even among healthcare professionals, there is little awareness of CB-PTSD [79, 89], so that women affected by CB-PTSD must also be concerned that their service providers will not be able to deal with their symptom adequately. Hence, it can be assumed that stigma is a greater barrier for women with CB-PTSD than it is for women with gPTSD.

Regarding Subscale (2) Health Beliefs, no differences could be identified between women affected by CB-PTSD or gPTSD and non-affected women. One of the items of this subscale (“I would rather talk to friends or family about my problems”) was the highest rated item among all women of our sample, indicating that even non-affected women seem to prefer to talk to their social environment instead of seeking professional services for treatment and counseling, maybe due to the already mentioned overall lacking knowledge about appropriate services and their effectiveness for treating CB-PTSD and other mental health problems.

The results for Subscale (3) Instrumental Barriers showed that women suffering from CB-PTSD or gPTSD reported more instrumental barriers (e.g. lack of time for and lack of childcare during treatment and counseling) than non-affected women. These results could be interpreted as an indication that non-affected women cannot fully empathize with affected women and cannot anticipate instrumental barriers that women with CB-PTSD or gPTSD perceive since they were never really confronted with the need for actual help-seeking.

One aim of the study was to uncover possible differences between women with CB-PTSD and women with gPTSD. In the context of different types of barriers, it can be summarized that no significant group differences were found in the *type of barriers* between the two subgroups of postpartum PTSD, but group differences between non-affected women and women affected by CB-PTSD or gPTSD were found regarding the *type of barriers*. Thus, overcoming barriers, especially instrumental ones, needs to be addressed for both types of postpartum PTSD in general and overcoming barriers related to fears about treatment and stigmatization specifically for CB-PTSD.

###  Strengths and limitations

To the best of our knowledge, this is the first study with a large sample comparing the likelihood of and barriers to help-seeking between postpartum women affected by postpartum PTSD and women not affected by clinically relevant PTSD symptoms that considers the two types of postpartum PTSD, namely CB-PTSD and gPTSD. Thanks to a detailed recruitment strategy and a high response rate, our sample is comparable to other samples of postpartum women in Germany and other European countries on a descriptive level [25, 35, 67, 90, 91]. Thus, valuable conclusions can be drawn from our results in order to improve access to appropriate counseling or treatment services of postpartum women who are affected by clinically relevant symptoms of CB-PTSD or gPTSD. Furthermore, the criteria for group allocation to the group of women affected by CB-PTSD allowed us to include women with highly stress causing birth experiences, even if they did not explicitly affirm the A criterion. Another strength of our study is that we primarily examined attitudes and intentions regarding help-seeking by assessing the likelihood of help-seeking. In contrast to simple comparisons of prevalence rates with utilization rates, knowledge about attitudes and intentions enables us to determine basic motivational features of help-seeking. Our study revealed that not attitudes about help-seeking hindered the process of help-seeking among postpartum women affected by clinically relevant symptoms of CB-PTSD or gPTSD, but specific barriers did. These could be reduced through targeted measures (see clinical implications).

Despite the aforementioned strengths of our study, it is important to address some limitations which may restrict the generalizability of our results. First, some requirements for statistical analyses were violated, which may restrict the generalizability of our results: Regarding the two ANCOVAs, residuals were not normally distributed and the covariates were not homogenously distributed across the symptom groups. The latter also applied to the MANCOVA. Furthermore, there was no homogeneity of error variance for Subscale (3) Instrumental Barriers.

Second, data were collected through telephone interviews and thus could be influenced by social desirability [92, 93]. Moreover, this assessment method contributes to a selection bias, as particularly severely affected women may not have had the capacity or resources to conduct a one-hour long telephone interview. Furthermore, results of the reliability analyses were low or questionable regarding the barrier subscales (2) Health Beliefs (α = 0.64) and (3) Instrumental Barriers (α = 0.58) and the corresponding publication describing the development of this measure is not yet published [84]. Thus, the results of these scales should be interpreted with caution.

## Research implications

Due to the small size of the group of women who were affected by comorbid symptoms of CB-PTSD and gPTSD, future studies should examine specific characteristics regarding the likelihood of and barriers to help-seeking of postpartum women with comorbid symptoms of CB-PTSD and gPTSD. As CB-PTSD may negatively affect the mother’s and child’s life beyond the period of 6 months postpartum chosen in this study [23–25], it would also be interesting to gain knowledge about the course of symptoms over time through follow-up interviews. Furthermore, research points to the widely spread comorbidity of CB-PTSD and gPTSD with PPD [94, 95]. That is why comorbid symptoms of these two disorders in the postpartum period and their links to the likelihood of and barriers to help-seeking should be examined.

### Clinical implications

The results of our study, particularly the identified disorder-specific barriers in help-seeking behavior of women with CB-PTSD, gPTSD, and women who are not affected by clinically relevant symptoms of these two disorders, imply that a targeted reduction of barriers such as stigma, instrumental factors, and knowledge and awareness gaps regarding CB-PTSD is key to improve access to appropriate services.

This is in line with central statements of a recently published expert review by Horsch et al. [96] that offers various recommendations for the implementation of preventive measures at the level of health care professionals as well as at the level of policy making in order to improve access to treatment and counseling services for postpartum women with CB-PTSD. These measures should include awareness campaigns, e.g. through information events in the context of pregnancy, where knowledge about postpartum mental health problems, and in particular about CB-PTSD or gPTSD, could be spread among perinatal women. This would better prepare them to recognize mental health problems after childbirth. Furthermore, prevention measures should also reach out to healthcare professionals in order to train them to screen for signs of PTSD symptoms after childbirth, e.g. during postnatal check-ups. This allows an early detection of symptoms of CB-PTSD or gPTSD and could reduce the fear of stigmatization as it would be part of routine examinations without the women having to take the initiative to seek help themselves. In order to further reduce stigma related barriers, offers that are incorporated in routine postpartum care examinations and integrated screenings for postpartum mental health problems seem to be crucial as also advocated for by Ayers et al. [97]. This should be combined with taking instrumental barriers into account, e.g. through integrated childcare options during maternal service utilization, sufficient offers in rural areas, or financial subsidies.

In addition, health professionals should render the birth as less traumatic as possible. To ensure that they are sufficiently trained for this, educational trainings about CB-PTSD and other postpartum mental health problems would be useful, as already in practice in some European countries (e.g. France, Netherlands) according to Thomson et al. [98]. With 740,000 childbirths in Germany every year [99] and therefore around 46,000 women affected by any type of postpartum PTSD, investing in preventive measures can avoid high financial burdens for society caused by treatment costs [100].

## Conclusions

This study aimed to generate knowledge about how to improve access to counseling or treatment options for postpartum women affected by symptoms of postpartum PTSD. To the best of our knowledge, this is the first study to focus on both layers of postpartum PTSD, namely CB-PTSD and gPTSD, in the context of help-seeking behavior of postpartum women. Postpartum women affected by symptoms of CB-PTSD, gPTSD, and women without clinically relevant symptoms of these two mental health problems did not differ significantly regarding the likelihood of help-seeking. Equally, the symptom severity of these two mental health problems could not predict the likelihood of help-seeking.

Overall group differences were found in the context of different types of barriers indicating that women affected by CB-PTSD or gPTSD reported more barriers in the help-seeking process than non-affected women. A special characteristic of the group of women with CB-PTSD seems to be that they are even more affected by fears about treatment characteristics and stigma than women with gPTSD. Thus, efforts should be made to reduce stigma (e.g. through awareness campaigns both among the general population as well as among perinatal mothers, and health professionals).

## Supplementary Information

Supplementary Material 1.

## Data Availability

The dataset presented in this article is not publicly available because of legal and ethical constraints. Public sharing of participant data was not included in the informed consent of the study. Requests to access the datasets should be directed to the project managers and principal investigators Susan Garthus-Niegel or Julia Schellong.

## Abbreviations

Abbreviation: Definition
PTSD: Posttraumatic stress disorder
CB-PTSD: Childbirth-related posttraumatic stress disorder
gPTSD: General posttraumatic stress disorder
PPD: Postpartum depression
PAD: Postpartum anxiety disorders
INVITE: INtimate partner Violence Treatment PrEferences
IPV: Intimate partner violence
City BiTS: City Birth Trauma Scale
PC-PTSD-5: Primary Care Posttraumatic Stress Disorder Screen for DSM-5
ANCOVA: Analysis of covariance
MLRs: Multiple linear regression analyses
MANCOVA: Multivariate analysis of covariance

## Footnotes

1 The term ‘CB-PTSD and/or gPTSD’ includes women with CB-PTSD, gPTSD and women with comorbid symptoms of CB-PTSD and gPTSD. If the term ‘CB-PTSD or gPTSD’ is mentioned, the comorbid group is not included. The distinction applies to the further explanations in this study.

2 In this and subsequent analyses for group comparison, women with comorbid CB-PTSD and gPTSD (*n* = 15) were excluded due to small sample size.
