# Supplementary material for: Access and barriers to treatment and counseling for postpartum women with and without symptoms of (CB-)PTSD within the cross-sectional study INVITE
Source: BMC Pregnancy Childbirth. 2026 Jan 24;26:93. doi: 10.1186/s12884-026-08660-x (PMC12849266; doi:10.1186/s12884-026-08660-x)
Supplement: Supplementary file 1 — Supplementary Material 1. [file 12884_2026_8660_MOESM1_ESM.docx]

**Supplemental material**

**Table A1**

*Differences between the Symptom Groups in the Likelihood of Help-Seeking Controlling for Confounders*

| Source | df | F | *p* | partial η² |
| --- | --- | --- | --- | --- |
| Corrected Model | 5 | 8.462 | < .001*** | .011 |
| Constant | 1 | 339.089 | < .001*** | .081 |
| Symptom groups ^a^ | 2 | 0.487 | .615 | .000 |
| Maternal age | 1 | 7.729 | .005** | .002 |
| Residence ^b^ | 1 | 2.583 | .108 | .001 |
| Income | 1 | 23.231 | < .001*** | .006 |
| Error | 3823 |  |  |  |
| Total | 3829 |  |  |  |
| Corrected Total | 3828 |  |  |  |

*Note.* Confounders = maternal age, duration of residence in Germany, and income.

^a^ symptom groups = non-affected, CB-PTSD, gPTSD, ^b^ duration of residence in Germany.

**p* < .05. ***p* < .01. ****p* < .001.

**Table A2**

*Multiple Linear Regression Results for Symptom Severity of CB-PTSD or gPTSD and the Likelihood of Help-seeking, Controlling for Confounders*

|  | Model | B | SE | *p* | 95% CI | ΔR² |
| --- | --- | --- | --- | --- | --- | --- |
| 1a | Constant | 7.954 | .044 | < .001**^***^** | [7.867, 8.038] | .000 |
|  | City BiTS sum | − .007 | .005 | .224 | [-.017, .004] |  |
| 2a | Constant | 6.657 | .338 | < .001**^***^** | [5.957, 7.335] | .006 |
|  | City BiTS sum | − .003 | .006 | .553 | [-.014, .008] |  |
|  | Maternal age | **.022** | .007 | .004^**^ | [.008, .036] |  |
|  | Residence ^a^ | .052 | .058 | .398 | [-.056, .170] |  |
|  | Income | **.079** | .025 | .002^**^ | [.030, .132] |  |
| 1b | Constant | 7.633 | .111 | < .001^**^ | [7.426, 7.911] | .002 |
|  | gPTSD severity | .033 | .020 | .104 | [-.006, .070] |  |
| 2b | Constant | 6.176 | .624 | < .001^**^ | [4.885 7.426] | .011 |
|  | gPTSD severity | .041 | .021 | .050 | [.002, .080] |  |
|  | Maternal age | .020 | .015 | .187 | [-.008, .052] |  |
|  | Residence ^a^ | .073 | .097 | .435 | [-.115, .267] |  |
|  | Income | **.131** | .053 | .010^*^ | [.026, .233] |  |

*Note*. City BiTS sum = CB-PTSD symptom severity (0–60), gPTSD severity = gPTSD symptom severity scale measured among women with at least one gPTSD symptom in the last month (0–20), Confounders = maternal age, duration of residence in Germany, income, SE*=*standard error, based on 1,000 bootstrap samples, CI = confidence interval. Significant multiple regression coefficients are printed in bold.

1a & 2a: Regression analysis a with symptom severity of CB-PTSD.

1b & 2b: Regression analysis b with symptom severity of gPTSD.

^a^ duration of residence in Germany.

**p* < .05. ***p* < .01. ****p* < .001.

**Table A3**

*Differences between the Symptom Groups in the Barriers Sum Score Controlling for Confounders*

| Source | df | F | *p* | partial η² |
| --- | --- | --- | --- | --- |
| Corrected Model | 7 | 19.785 | < .001*** | .035 |
| Constant | 1 | 448.432 | < .001*** | .105 |
| Symptom groups ^a^ | 2 | 2.720 | .066 | .001 |
| Groups*maternal age | 2 | 2.566 | .077 | .001 |
| Maternal age | 1 | 8.501 | .004** | .002 |
| Residence ^b^ | 1 | 29.074 | < .001*** | .008 |
| Income | 1 | 23.529 | < .001*** | .006 |
| Error | 3815 |  |  |  |
| Total | 3823 |  |  |  |
| Corrected Total | 3822 |  |  |  |

*Note.* Confounders = maternal age, income, duration of residence in Germany.

^a^ symptom groups = non-affected, CB-PTSD, gPTSD, ^b^ duration of residence in Germany.

**p* < .05. ***p* < .01. ****p* < .001.

**Table A4**

*Differences between the Symptom Groups in the Subscales of Barriers to Help-Seeking*

*Controlling for Confounders*

| Source | Dependent variables | df | F | *p* | partial η² |
| --- | --- | --- | --- | --- | --- |
| Model (corrected) | Subscale 1 | 5 | 15.548 | < .001*** | .020 |
|  | Subscale 2 | 5 | 18.835 | < .001*** | .024 |
|  | Subscale 3 | 5 | 20.919 | < .001*** | .027 |
|  |  |  |  |  |  |
| Constant | Subscale 1 | 1 | 734.859 | < .001*** | .163 |
|  | Subscale 2 | 1 | 1191.652 | < .001*** | .239 |
|  | Subscale 3 | 1 | 916.635 | < .001*** | .195 |
|  |  |  |  |  |  |
| Symptom groups ^a^ | **Subscale 1** | **2** | **12.468** | **< .001***** | **.007** |
|  | Subscale 2 | 2 | 1.105 | .331 | .001 |
|  | **Subscale 3** | **2** | **17.978** | **< .001***** | **.009** |
|  |  |  |  |  |  |
| Maternal age | Subscale 1 | 1 | 31.930 | < .001*** | .008 |
|  | Subscale 2 | 1 | 36.205 | < .001*** | .009 |
|  | Subscale 3 | 1 | .083 | .773 | .000 |
|  |  |  |  |  |  |
| Residence ^b^ | Subscale 1 | 1 | 4.583 | .032* | .001 |
|  | Subscale 2 | 1 | 21.195 | < .001*** | .006 |
|  | Subscale 3 | 1 | 27.747 | < .001*** | .007 |
|  |  |  |  |  |  |
| Income | Subscale 1 | 1 | 3.228 | .072 | .001 |
|  | Subscale 2 | 1 | 16.350 | < .001*** | .004 |
|  | Subscale 3 | 1 | 26.767 | < .001*** | .007 |
|  |  |  |  |  |  |
| Error | Subscale 1 | 3787 |  |  |  |
|  | Subscale 2 | 3787 |  |  |  |
|  | Subscale 3 | 3787 |  |  |  |
|  |  |  |  |  |  |
| Total | Subscale 1 | 3793 |  |  |  |
|  | Subscale 2 | 3793 |  |  |  |
|  | Subscale 3 | 3793 |  |  |  |

*Note.* Subscale 1 = Fears about Treatment and Stigmatization, Subscale 2 = Health Beliefs, Subscale 3 = Instrumental Barriers. Confounders = maternal age, duration of residence in Germany, income. Significant group differences regarding the respective subscale are printed in bold.

^a^ symptom groups = non-affected, CB-PTSD, gPTSD, ^b^ duration of residence in Germany

**p* < .05. ***p* < .01. ****p* < .001.
